# Supplementary material for: Powassan Virus Induces Structural Changes in Human Neuronal Cells In Vitro and Murine Neurons In Vivo
Source: Pathogens. 2022 Oct 21;11(10):1218. doi: 10.3390/pathogens11101218 (PMC9609669; doi:10.3390/pathogens11101218)
Supplement: Supplementary file 1 [file pathogens-11-01218-s001.zip › pathogens-1914847-supplementary.pdf]

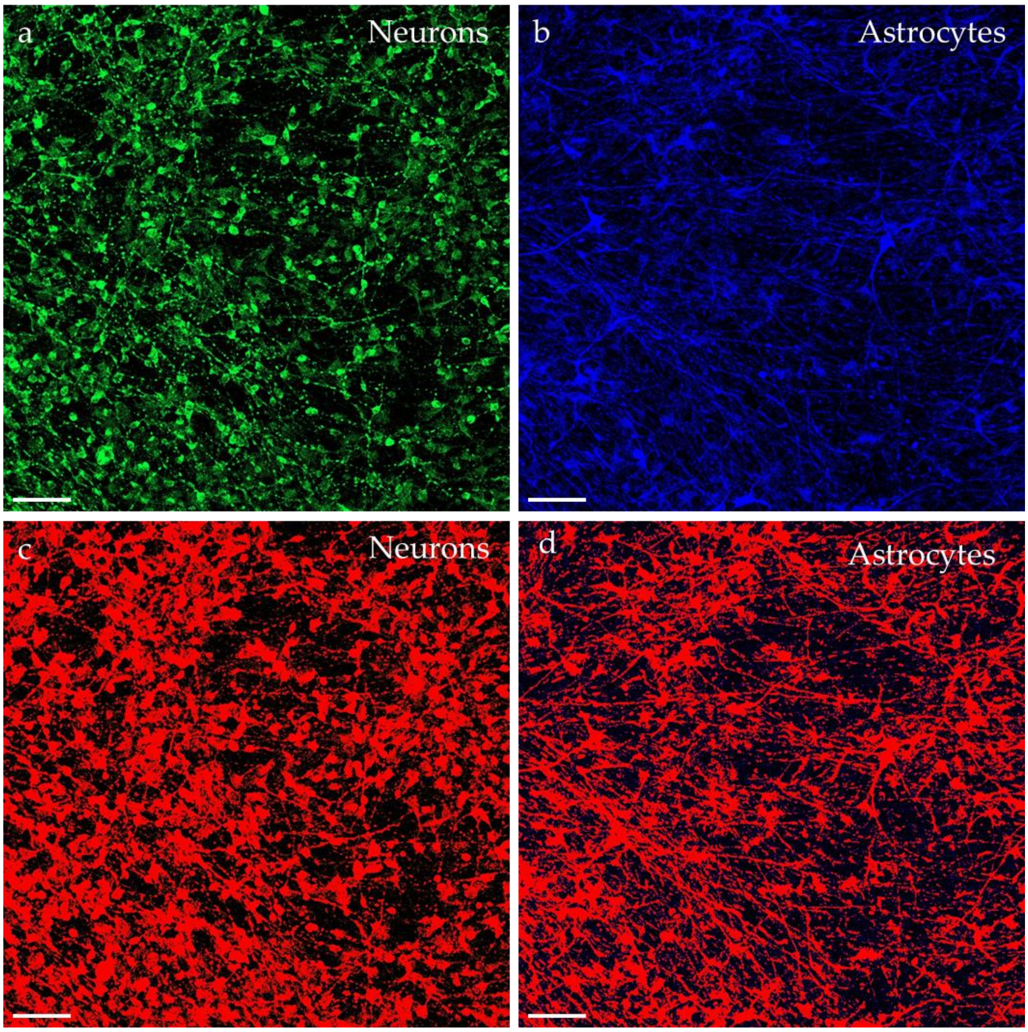

Figure S1: Composition of hNSC-derived neuron/astrocyte co-cultures. Differentiated hNSC-derived neuron/astrocyte co-cultures were fixed after 72 hours. **(a)** Neurons were stained using an anti-MAP2 (green) antibody, while **(b)** astrocytes were stained using an anti-GFAP (blue) antibody. Images were taken using confocal microscopy as previously described at 10x magnification. **(c, d)** Z-stacks were combined into a max projection image and then threshholded using FIJI (red). The total area was determined and used to calculate percentages of neurons and astrocytes. Scale bars represent 50 μm.

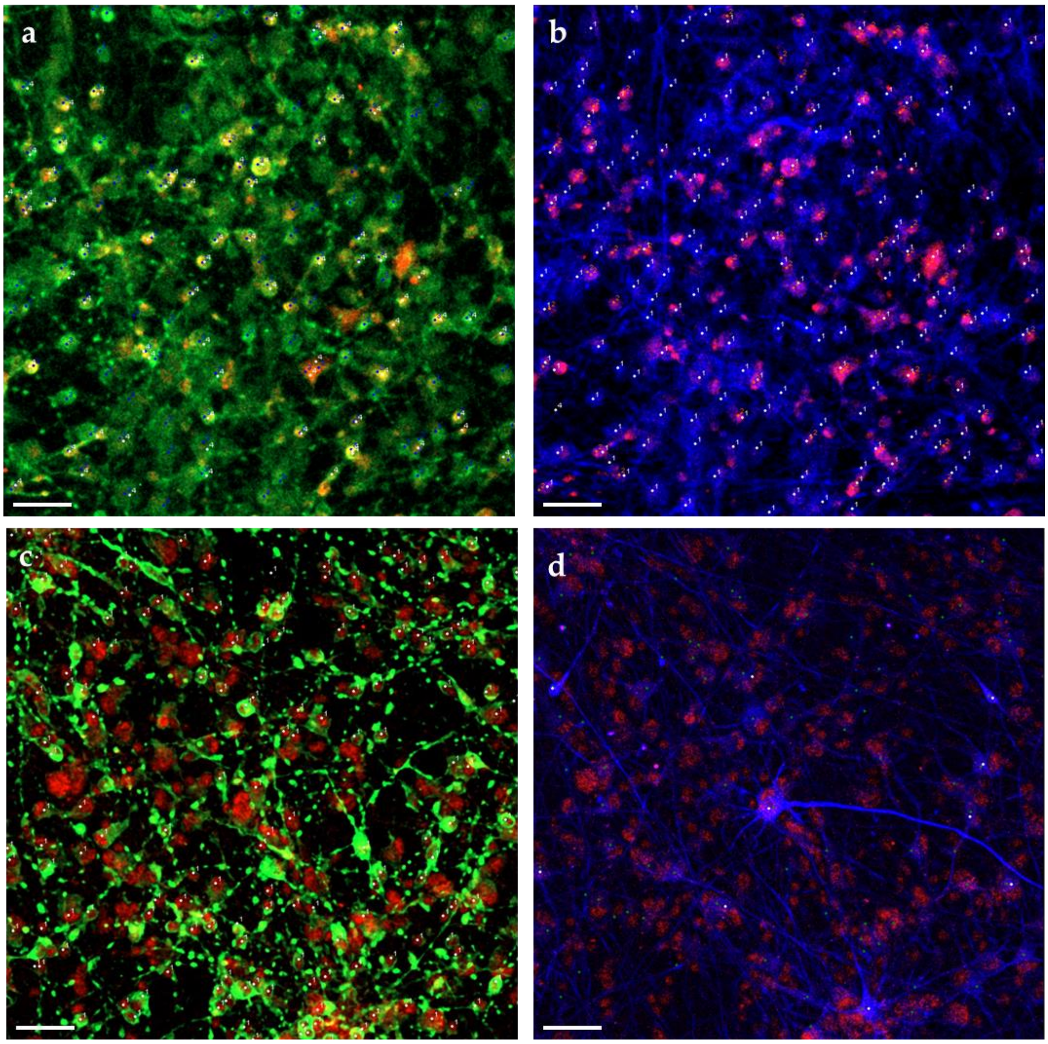

Figure S2. Quantification of infected hNSC-derived neuron/astrocyte co-cultures. Differentiated hNSC-derived neuron/astrocyte co-cultures were infected with 1.0 MOI POWV or WNV and fixed after 72 hours. Neurons were stained with an anti-MAP2 antibody (green), astrocytes were stained using an anti-GFAP (blue) antibody, POWV was stained using was stained using rabbit polyclonal anti-POWV (red) antibodies and WNV was stained using rabbit polyclonal anti-WNV (red) antibodies. Images were taken using confocal microscopy as previously described at 20x magnification. Z-stacks were combined into a max projection image and cells were counted by hand using Fiji Cell Count plugin. (**a**) POWV-infected neurons. (**b**) POWV-infected astrocytes. (**c**) WNV-infected neurons. (**d**) WNV-infected astrocytes. Scale bars represent 20 μm.

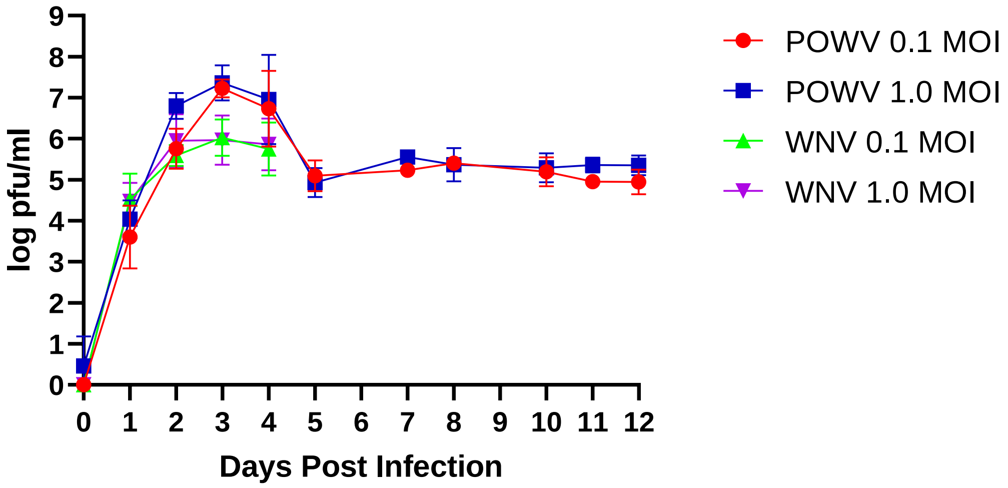

Figure S3. Growth kinetics of POWV and WNV in hNSC-derived neuron/astrocyte co-cultures. Differentiated hNSC-derived neuron/astrocyte co-cultures were infected with either 0.1 or 1.0 MOI of POWV or WNV. Cultures were sampled individually every 24 hours for up to 12 days and virus titers determined by plaque assay to establish viral growth kinetics. (n=9 days 0-4; n=3 days 5-12).

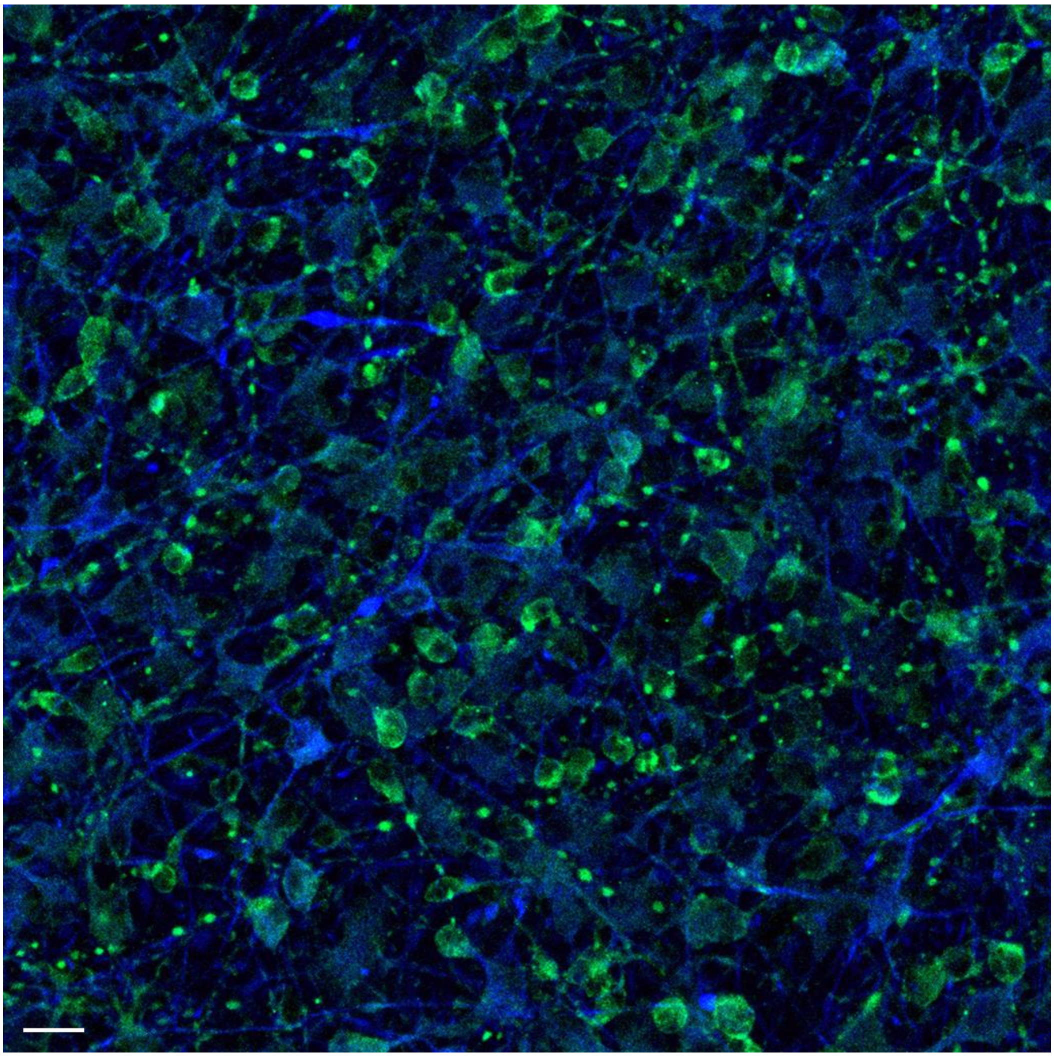

Figure S4: MOCK Infected hNSC-derived neuron/astrocyte co-cultures. Differentiated neuron/astrocyte co-cultures were fixed after 72 hours, and neurons stained using anti-MAP2 (green) antibodies, while astrocytes were stained using anti-GFAP (blue) antibodies. Image was taken using confocal microscopy as previously described at 20x magnification. Z-stacks were combined into a max projection image. Scale bar represents 10 μm.
